# Case Report: Carbonic anhydrase inhibitor brinzolamide dramatically improved the morphology and also function of a patient with RS1 mutation

**DOI:** 10.3389/fphar.2025.1706488

**Published:** 2025-11-04

**Authors:** Yue Ren, Shu Liu, Jia Rong, Di Wang, Miao Diao, Yaqi Zhang, Shimiao Tian, Mingxin Shang, Chuqiao Song, Yan Guan, Zhuoshi Wang, Jijing Pang

**Affiliations:** ^1^ Shenyang He Eye Specialist Hospital, Shenyang, China; ^2^ Institute of Innovation Research for Precision Medical Treatment, He University, Shenyang, China; ^3^ Liaoning Provincial Innovation Center of Ophthalmology, Shenyang, China

**Keywords:** RS1 gene, X-linked, retinoschisis, carbonic anhydrase inhibitor, foveal thickness

## Abstract

X-linked retinoschisis (XLRS) is an inherited retinal disease caused by mutations in the *RS1* gene, which encodes retinoschisin, a protein essential for maintaining the retinal structure during development. Here, we report the therapeutic evidence in a male patient diagnosed with X-linked retinoschisis (XLRS) before and after topical treatment with a carbonic anhydrase inhibitor (CAI), brinzolamide. XLRS-like typical retinal morphology and visual function before treatment and novel improvements gradually from one to 3 months following treatment were observed; then a recurrence of foveal retinoschisis and decreased retinal function occurred because the use of brinzolamide eye drops was disrupted by months-long recurrent upper respiratory tract infections; eventually retinal improvement after resuming brinzolamide were found again, confirming that brinzolamide eye drops could reduce retinoschisis and improve visual acuity. This individualized “dechallenge–rechallenge” evidence chain provides direct supports for the brinzolamide to be the cause in controlling XLRS progression rather than the natural occurring in the course of the disease itself.

## 1 Introduction

X-linked retinoschisis (XLRS, OMIM#312700) is a bilateral retinal degenerative disorder that primarily affects males, following an X-linked recessive inheritance pattern. Female carriers typically do not exhibit fundus abnormalities, although rare cases of heterozygotes with clinical features have been reported (Pineda-Garrido et al., 2022; Ranabhat et al., 2022). XLRS is characterized by the high variability in clinical manifestation, symptoms such as central vision loss typically emerge during school age; rarely, they manifest in infancy (George et al., 1995). The main macular changes include macular holes (about 78%–88%), macular atrophy (about 10%–16%), and a small number of patients have normal macular morphology; about 50% of patients also have peripheral retinal changes, including flat retinal tears (Chen et al., 2020; Orès et al., 2018; Xiao et al., 2023). At present, *RS1* (retinoschisin 1) is the only causative gene definitively linked to the disease. The *RS1* gene is located at Xp22.1-p22.2, spans 32,421 base pairs and comprises six exons. It encodes retinoschisin, which is expressed and secreted by the photoreceptors and bipolar cells. Studies have shown that retinoschisin is critical for retinal cell-cell adhesion and for the signaling processes at photoreceptor-bipolar synapses. Pathogenic *RS1* variants have been demonstrated to cause retinal structural deterioration, leading to the formation of cystic cavities in the macular area and peripheral retinoschisis, ultimately impairing vision. Currently, there are no effective treatments available for XLRS in clinical practice (Reid et al., 2003; Vijayasarathy et al., 2022), however, some studies and a few trials have shown that carbonic anhydrase inhibitors (CAIs) can reduce the volume of macular schisis cavities, improve visual acuity, and reduce the incidence of complications such as macular atrophy and retinal detachment in XLRS patients (Ambrosio et al., 2021; Apushkin and Fishman, 2006; Testa et al., 2019; Wey et al., 2023). Studies speculate that CAIs do not directly treat XLRS; instead, they acidify the subretinal space, lower the retinal standing potential, and enhance retinal adhesiveness, thereby driving fluid out of the retina, shrinking cystic cavities, and possibly promoting cellular apposition (Testa et al., 2019; Thobani and Fishman, 2011; Wolfensberger, 1999). This study reports the clinical progression of a patient with XLRS following topical administration of a carbonic anhydrase inhibitor (CAI).

## 2 Case description

### 2.1 Gene sequencing results

**FIGURE 1:**
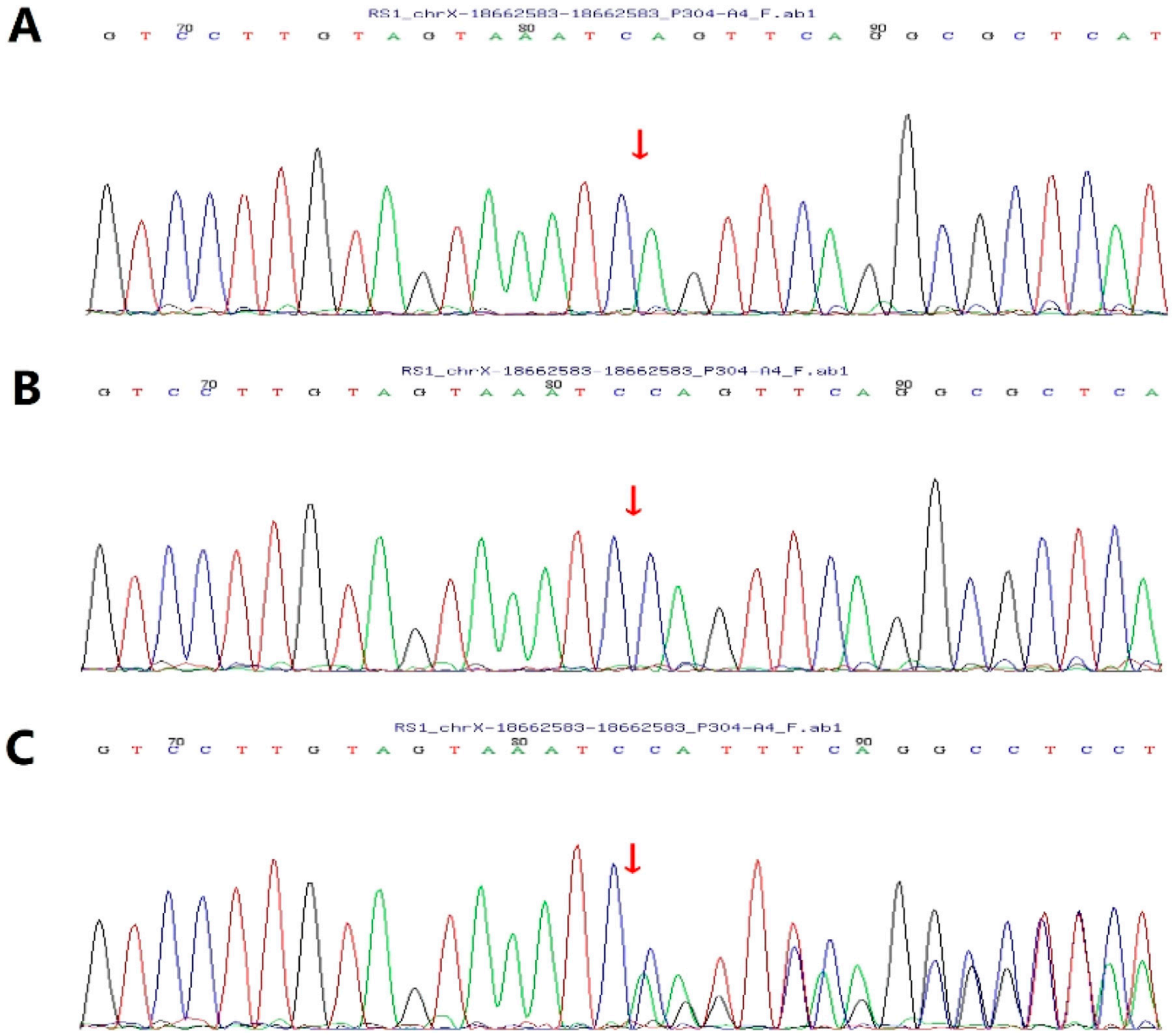
Sanger validation of c.489delC (p. Trp163Ter). **(A)** Patient: hemizygous; **(B)** The patient’s father: wild-type; **(C)** The patient’s mother: heterozygous.

This study reports a Chinese boy who first visited our hospital at 7 years of age. Genetic testing revealed a hemizygous, previously unreported nonsense mutation in the *RS1* gene: c.489delC (p. Trp163Ter) in exon 5. This mutation was inherited from the patient’s mother (Figure 1). It is noteworthy that while pathogenic variants leading to a termination codon at the same amino acid position (tryptophan 163) have been documented (Li et al., 2007), this specific nucleotide deletion in our patient is novel.

### 2.2 Clinical manifestation

#### 2.2.1 Baseline

**FIGURE 2:**
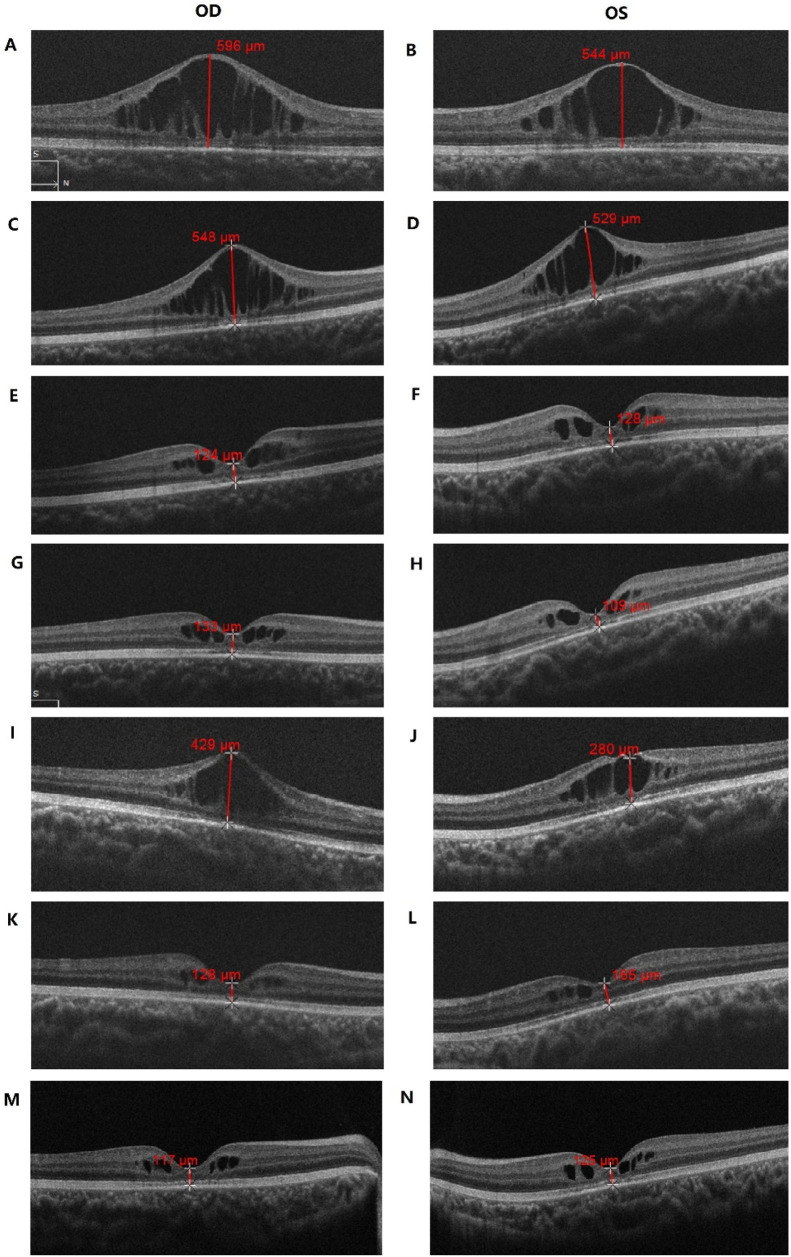
OCT examination results of the patient. **(A,B)** Macular OCT of the patient before treatment; **(C–H)** Macular OCT after 1-**(C,D)**, 2-**(E,F)** and 3-**(G,H)** months of topical brinzolamide, showing progressive improvement in macular schisis; **(I,J)** Macular OCT 3 months after brinzolamide was stopped, with worsening macular schisis; **(K–N)** Macular OCT after resuming brinzolamide for 6-**(K,L)** and 18- **(M,N)** months, demonstrating improvement in macular schisis.

Bilateral visual impairment had been noted at his routine preschool examination at age 4 years, but no diagnostic or therapeutic details were available. At presentation to us optical coherence tomography (OCT) showed that the lesion involved the inner nuclear layer (INL) and outer plexiform layer (OPL) surrounding the fovea, with bridging strands within the schisis. The foveal thickness (FT) was measured at 596 µm in the right eye (OD) and 544 µm in the left eye (OS) (Figures 2A,B). Best-corrected visual acuity (BCVA) was 0.5 logMAR in both eyes (OU).

#### 2.2.2 Initial treatment and response

Topical CAI therapy with brinzolamide eye drops 1% (Azopt, Alcon Laboratories, United Kingdom) twice daily was started; The patient had no history of prior ocular medication.

One month into CAI treatment, OCT revealed a slight reduction in macular-cyst height, with FT 548 µm OD and 529 µm OS (Figures 2C,D); BCVA improved to 0.4 logMAR OU.

Two months after starting therapy, OCT showed that the retinoschisis had almost completely resolved, leaving only minimal cystic maculopathy. FT had fallen markedly to 124 µm OD and 128 µm OS (Figures 2E,F), and BCVA remained 0.4 logMAR OU.

After continuing CAI for 3 months, OCT demonstrated macular morphology similar to that observed 1 month earlier, with minimal cystic change (Figures 2G,H); BCVA was 0.4 logMAR OD and 0.3 logMAR OS.

#### 2.2.3 Treatment interruption and relapse

During the next scheduled 3-month follow-up period the child experienced three recurrent intercurrent upper respiratory tract infections caused by different pathogens—characterized by fever, cough and rhinorrhea. The substantial distance from the hospital (∼700 km) and recurrent childhood illnesses led to a lapse in follow-up and the inadvertent discontinuation of therapy.

At the 6-month observation point—three months after stopping drops—retinoschisis had worsened: FT 429 µm OD and 280 µm OS (Figures 2I,J), BCVA 0.5 logMAR OU.

#### 2.2.4 Re-treatment and long-term outcome

Three months after the treatment was stopped, the patient resumed brinzolamide. Based on his condition, we adjusted the dosage to three times daily.

At the 12-month observation visit—6 months after resuming treatment—OCT showed marked reduction of cystic maculopathy in the INL surrounding the fovea, with FT 128 µm OD and 165 µm OS (Figures 2K,L); BCVA had improved further to 0.3 logMAR OU. Since restarting brinzolamide the patient has remained stable. At the 18-month observation visit BCVA was maintained at 0.3 logMAR OU.

At the 24-month observation visit—18 months after therapy resumption—OCT disclosed only minimal INL schisis around the fovea, while macular morphology remained stable: FT 117 µm OD and 125 µm OS (Figures 2M,N), BCVA still 0.3 logMAR OU.

During the follow-up period, the patient’s intraocular pressure (IOP) remained within the normal range all the time. BCVA, FT and IOP of the patients during the follow-up are detailed in Table 1.

**TABLE 1:** Application of brinzolamide eye drops and table of changes in BCVA, FT and IOP.

Treatment stage	Medication status	BCVA		FT		IOP	
		VD (logMAR)	VS (logMAR)	FT-OD (µm)	FT-OS (µm)	R (mmHg)	L (mmHg)
Initial visit to our hospital	Brinzolamide eye drops started	0.5	0.5	596	544	14	14
1-Month observation	1 Month after medication initiation	0.4	0.4	548	529	12	13
2-Month observation	2 Months after medication initiation	0.4	0.4	124	128	17	16
3-Month observation	3 Months after medication initiation	0.4	0.3	133	109	12	11
3-Month drug discontinuation	Brinzolamide eye drops restarted	0.5	0.5	429	280	12	17
12-Month observation	6 Months after medication restart	0.3	0.3	128	165	12	12
18-Month observation	12 Months after medication restart	0.3	0.3	No data	No data	16	17
24-Month observation	18 Months after medication restart	0.3	0.3	117	125	13	14

## 3 Discussion

In this case, the patient received local application of CAI for 24 months, without any side effects or adverse reactions. During CAI follow-up, after the schisis had improved and stabilized, the patient developed symptoms of the recurrent intercurrent upper respiratory tract infections, the patient discontinued CAI and received symptomatic treatment for upper respiratory tract infections. It was found that the macular foveal schisis worsened after stopping the CAI, and the symptoms improved again after continuing to add CAI. Considering all these factors, whether the infection of the patient can exacerbate the condition of XLRS still requires further medical record observation and animal experiments to determine the factors affecting foveal schisis. However, what we know is that when CAI was reinstated, the patient’s symptoms improve significantly and the macular morphology returns. It could reduce the height of the cystoid macular elevation, thereby maintaining the tissue morphology of the macula. This result is consistent with the effect of CAI application in our other similar patients. However, as those patients did not recur following treatment, we cannot prove whether the improvement was directly caused by the use of CAI or was a natural improvement of the disease itself.

A previously reported patient had the same amino acid alteration (p. Trp163Ter) due to a different nucleotide change (c.488delG). In contrast, our patient harbored a novel c.489delC mutation (Li et al., 2007). Both probands presented at age 4 years with isolated macular schisis and no evidence of peripheral retinoschisis. Notably, the historical case still exhibited persistent foveal schisis at 9 years of age without any documented intervention, while our patient demonstrated marked resolution of macular schisis by the final visit at 9 years. This comparison provides indirect evidence that CAI treatment may confer beneficial morphological outcomes in RS1-associated retinoschisis, even when the underlying genotype is identical. During treatment, not only did the macular morphology improve, but the patient’s visual acuity also increased markedly, thus proving that macular structure has a significant impact. We speculate that normal macular tissue morphology can not only facilitate visual development but also reduce developmental visual anomalies such as amblyopia. We further infer that well-preserved macular architecture may enhance visual recovery after gene therapy.

The *RS1* gene encodes retinoschisin, a protein that plays a crucial role in maintaining both the structural integrity and functional capabilities of the retina. Mutations in the *RS1* gene can disrupt or terminate retinoschisin synthesis, resulting in schisis of the inner retinal layers. Clinically, XLRS in males presents as bilateral macular and possibly peripheral retinoschisis. Complications may include retinal detachment and hemorrhage within the schisis cavities or the vitreous cavity. If no intervention is made, XLRS usually leads to macular atrophy in the fifth or sixth decade of life. Currently, the prevailing therapeutic approach for XLRS is gene augmentation therapy, which delivers the functional *RS1* gene to affected retinal cells. However, two related clinical trials conducted in the United States and Europe have reported that the vision recovery after treatment did not achieve the expected endpoints in phase I/II trials (Ku et al., 2023; Pennesi et al., 2022). While awaiting gene therapy, clinicians have conducted extensive research, including the use of CAIs to regulate disease progression. Nevertheless, the efficacy of CAIs in treating XLRS remains uncertain (Ambrosio et al., 2021; Apushkin and Fishman, 2006; Testa et al., 2019; Wey et al., 2023; Khandhadia et al., 2011). In recent years, both topical and oral treatments have been reported to reduce the height of macular lesions, potentially benefiting patients with XLRS who present with cystic macular lesions and mid-peripheral retinoschisis. There is evidence to suggest (Ambrosio et al., 2021) that early systemic or local administration of CAI may confer benefits to the retina of XLRS patients, potentially by reducing intraocular fluid accumulation, promoting contraction of the subretinal space, reducing retinal thickness, and supporting restoration of retinal anatomy, thereby improving vision and potentially enhancing macular function; Although CAI cannot directly treat XLRS, it has been shown to reduce retinal fluid by inhibiting the catalytic conversion of carbon dioxide and water into carbonates (which are subsequently converted into bicarbonates). This process reduces the volume of cystic spaces and potentially promotes cell adhesion. Furthermore, metabolic acidosis, which has been demonstrated to reduce neural excitability, requires careful consideration. Inhibition of carbonic anhydrase leads to elevated carbon dioxide concentrations, thereby reducing gamma-aminobutyric acid (GABA) synthesis and causing extracellular glutamate accumulation. In the long term, even after discontinuing CAI therapy, the same chronic condition may persist, potentially leading to excitotoxicity and neurodegeneration. And there are currently no standardized clinical guidelines for treating XLRS with CAIs. Consequently, the long-term effects of CAI on the retina are of significant concern. In conclusion, this individualized “dechallenge–rechallenge” chain of evidence for drug discontinuation due to patient influenza in the present case directly supports brinzolamide as the cause of controlling XLRS progression rather than a naturally occurring cause during the course of the disease itself. However, there is a paucity of large samples and long-term clinical observations that would prove the necessity and safety of CAI in patients with XLRS. We will continue to summarise a large number of clinical data and recommend that patients with XLRS can first use CAI to maintain macular morphology and existing vision, so as to prepare for subsequent gene therapy.

## Data Availability

The corresponding author made the data utilized in the present investigation accessible to interested individuals upon a reasonable request.
